# *ASH1L* may contribute to the risk of Tourette syndrome: Combination of family‐based analysis and case–control study

**DOI:** 10.1002/brb3.2539

**Published:** 2022-03-20

**Authors:** Wenmiao Liu, Lulu Xu, Cheng Zhang, Lu Shen, Jicheng Dong, Han Zhang, Shiguo Liu, Fengyuan Che, Xueping Zheng

**Affiliations:** ^1^ Department of Medical Genetics The Affiliated Hospital of Qingdao University Qingdao China; ^2^ Department of Geriatric Medicine The Affiliated Hospital of Qingdao University Qingdao China; ^3^ Department of Neurology Linyi People's Hospital The Eleventh Clinical Medical College of Qingdao University Linyi China; ^4^ Qingdao Mental Health Center Qingdao China; ^5^ Department of Psychiatry Qingdao University Qingdao China

**Keywords:** *ASH1L*, haplotype relative risk, Tourette syndrome, transmission disequilibrium test

## Abstract

**Objective:**

Tourette syndrome (TS) is a childhood neurodevelopmental disorder caused by various genetic and environmental factors and presents with apparent genetic heterogeneity. As *ASH1L* potentially contributes to neurodevelopmental diseases, especially in TS, we aim to investigate the susceptibility of *ASH1L* on TS in the Chinese Han population.

**Methods:**

Three tag single nucleotide polymorphisms (SNPs) (rs5005770, rs12734374, and rs35615695) in *ASH1L* were screened in 271 TS nuclear family trios and 337 healthy subjects by the TaqMan assays real time. A case–control study combined with family‐based analysis was applied to study the genetic susceptibility of common variants of *ASH1L*.

**Results:**

The results revealed a significant over‐transmission of rs35615695 and rs5005770 (for rs35615695, transmission disequilibrium test, *χ* ^2^ = 57.375, *p* = .000, HHRR, *χ* ^2^ = 4.807, *p* = .028; for rs5005770, HRR, *χ* ^2^ = 4.116, *p* = .042, HHRR, *χ* ^2^ = 8.223, *p* = .004) in family‐based study. Furthermore, rs5005770 and rs35615695 still remained significant after Bonferroni correction (*p* < .017). However, the two SNPs (rs5005770 and rs35615695) were found not to be associated with TS in case–control study.

**Conclusions:**

Our study suggests that *ASH1L* may contribute to TS susceptibility in the Han Chinese population and involved in TS development as a risk factor.

## Graphical abstract

Tourette syndrome is a childhood neurodevelopmental disorder, caused by a variety of genetic and environmental factors and presents with obvious genetic heterogeneity. As *ASH1L* potentially contributes to neurodevelopmental diseases, especially in TS, we aim to investigate the susceptibility of *ASH1L* on TS in the Chinese Han population. Three tag single nucleotide polymorphisms (SNPs) (rs5005770, rs12734374, and rs35615695) in *ASH1L* were screened in 271 TS nuclear family trios and 337 healthy subjects by the TaqMan assays real time. A combination of case–control study with family‐based analysis was applied to study the genetic susceptibility of common variants of *ASH1L*. Our study suggests that *Ash1L* may contribute to TS susceptibility in the Han Chinese population and as a risk factor involved in the development of TS.

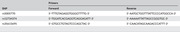

## 1 INTRODUCTION

Tourette syndrome (TS) is a neurodevelopmental disorder with the dysfunction of basal ganglia–cortical interaction. The prevalence of TS rises from 1/2000 that was thought to be in the 1980s, to 1%, a robust figure coming from community‐determined studies in schools and other settings (Stern, 2018). TS, characterized by multiple and chronic vocal and motor tics, is two to four times more common in males than in females. Most cases develop before adolescence and often at 5–7 years of age, and its peak severity is on average at 13 years (Browne et al., 2015; Robertson, 2015). A high rate of comorbidity occur in TS, including attention deficit hyperactivity disorder (ADHD) and obsessive‐compulsive disorder (OCD), which are the two most strongly related conditions (Freeman et al., 2000; Robertson, 2006; Robertson et al., 2015). Although the etiology of TS has not been fully illuminated, the genetic predisposition hypothesis is widely and preferentially considered (Ünal & Akdemir, 2016). Evidence based on the familial aggregation studies showed that the risk for first‐degree relatives was significantly higher than that for individuals in the general population (Pauls et al., 1991, 1981, 2014). In twin studies, 53−56% of monozygotic twins were concordant for TS, whereas only 8% of dizygotic twins were concordant for TS (Price et al., 1985). Although these studies indicated that genetic factors play the significant role in TS etiology, the exact mechanisms remain unknown.

Whole‐exome sequencing (WES) of 100 TS trios was performed and de novo mutations analysis and rare variant‐transmission disequilibrium test (TDT) were also conducted to identify a risk gene, *ASH1L*, and to reveal mutations in *ASH1L* confer susceptibility to TS in our previous study (S. Liu et al., 2020). The *ASH1L* is located on 1q22, containing 33 exons, of which mRNA length is 11784 bp encoding 2964 amino acids. ASH1L is a large multidomain protein that contains motifs involved in chromatin remodeling including four AT hooks, a SET domain, a PHD finger, a bromodomain, a Bromo‐adjacent homology domain (BAH), and MYND ligand domains (Nakamura et al., 2000). As a highly conserved H3K4 methyltransferase, the *ASH1L* can inhibit NF‐kB and MAPK pathways to negatively regulate the secretion expression of *IL*‐6 and *TNF* triggered by TRL in macrophages protecting the body against endotoxic shock (Schuettengruber et al., 2011). Furthermore, *ASH1L* also plays an essential role in promoting the neural precursor cells' growth, development, polarization, migration, and synaptic growth (Gregory et al., 2007). Actually, *ASH1L* has been thought to play a pathogenic role in other more common neuropsychiatric disorders except for TS (Shen et al., 2019; Stessman et al., 2017; Wang et al., 2016).

In the context of our previous studies, we conducted further researches on well‐defined patient cohorts. This study first investigated the potential association between three single nucleotide polymorphisms (SNPs) (rs5005770, rs12734374, and rs35615695) in *ASH1L* and TS in the Chinese Han population. The combination of case–control study with family‐based analysis was applied to study the genetic susceptibility of common variants of *ASH1L* in 271 TS trios and 337 control subjects, which can significantly improve the testing strength and reliability of the correlation analysis.

## 2 MATERIALS AND METHODS

### 2.1 Subjects

The 271 TS nuclear family trios and 337 control subjects were selected from the Affiliated Hospital of Qingdao University. All patients with TS were diagnosed according to the criteria of the Diagnostic and Statistical Manual of Mental Disorders, 5th Edition (DSM‐V). We excluded patients with an uncertain diagnosis, organic brain diseases, genetic disorders, or a genetic history. Both the patients and their legal guardians agreed to participate in the study and signed the informed consent. We also obtained the approval of the Human Ethics Committee of the Affiliated Hospital of Qingdao University.

### 2.2 Selection of SNPs and genotyping

Venous blood samples (3–4 ml) were collected from the research objects and stored in the vacuum vasculature of EDTA anticoagulation. We isolated genomic DNA using TIANamp Blood DNA Kit (TIANGEN BIOTECH, Beijing, China) according to the direction from the peripheral blood samples. Three tag SNPs (rs5005770, rs12734374, and rs35615695) across the *ASH1L* region were selected from the HapMap CHB database. The rs35615695 was in intron 1, and rs5005770 was in intron 10. Rongve et al. (2019) have found that the rs12734374 was associated with dementia with Lewy Bodies (DLB), a neurodegenerative disorder. The rs5005770 and rs35615695 are important tag SNPs of ASH1L, and the clinical significance has not been reported. These three selected SNPs are all important tag SNPs in ASH1L in Chinese Han population.

Three TaqMan™ SNP Genotyping Assay Kit were purchased from Thermo Fisher for different tag SNPs. Then, the genetic genotyping of theses SNPs was performed via TaqMan allelic discrimination real‐time PCR. The real‐time polymerase chain reaction followed the following systems and conditions: system: TaqMan™ProAmp™Master Mix 12.5 µl, TaqMan™ SNP Genotyping Assay (20×) 1.25 µl, Genomic DNA 1 µl, nuclear‐free water 10.25 µl; conditions: pre‐read (60℃ for 30 s), initial denature/enzyme activation (95℃ for 5 min), 40 cycles of denature (95℃ for 15 s), anneal/extend (60℃ for 60 s), post‐read (60℃ for 30 s). The genotype of each sample could be identified by detecting the fluorescent signal from VIC‐ or FAM‐labeled probes in each cycle. The primer sequences used for each SNP are listed in Table 1.

**TABLE 1:** The forward and reverse primers of the three SNPs

SNP	Primers	
	Forward	Reverse
rs5005770	5′‐TTTGTAGAGGTGGGGTTTTG‐3′	5′‐AAAAATTATTAGCCGGGTGC‐3′
rs12734374	5′‐TGGATCACGAGGTCAGGAGATT‐3′	5′‐CTTCCCCTTTGCCTCTCCTT‐3′
rs35615695	5′‐CTAGTTACTCTGGAGGCTGAGATGAG‐3′	5′‐AGGTTGCAGTGAGCCGAGAT‐3′

### 2.3 Statistical analysis

The data were analyzed by statistical software SPSS 26.0. Firstly, we performed Hady–Weinberg (H–W) equilibrium test on control groups. The Person's chi‐square test was applied to assess the statistical significance of the allelic and genotypic distribution between cases and controls. Family data were analyzed by transmission disequilibrium test (TDT) and haplotype relative risk (HRR), as well as the haplotype‐based haplotype relative risk (HHRR). The HRR strategy compares allele frequencies in affected offspring with the frequency of parental alleles not transmitted to affected offspring. The TDT uses the distribution of marker alleles among families to test for association, while controlling for population heterogeneity. This test specifically considers parents who are heterozygous and evaluates the transmission frequency of the allele to affected offspring. Genotype and allele distributions were analyzed using chi‐square test, the TDT test being a McNemar chi‐square test. The 95% confidence intervals (CIs) and odds ratios (ORs) were used to show the relative risk degree. The *p* values < .05 suggested that the difference was statistically significant. In addition, the Bonferroni correction test was performed to improve the precision of the results that *p* values < .017 were thought to be significant.

## 3 RESULTS

### 3.1 The case–control study

All subjects were divided into case (271 TS individuals, 211 males and 60 females, mean age, 8.78 ± 3.20 years) and control groups (337 healthy subjects, 265 males and 72 females, mean age, 32.24 ± 7.59 years). The genotypic frequencies for three SNPs in control groups obeyed to HW equilibrium (rs5005770, *χ* ^2^ = .103, *p *= .749; rs12734374, *χ* ^2^ = .130, *p *= .718; rs35615695, *χ* ^2^ = 2.916, *p *= .088) (Table 2).

**TABLE 2:** The H–W equilibrium test for three SNPs on control groups

SNP	H–W equilibrium test	
	*χ* ^2^	*p*
rs5005770	.103	.749
rs12734374	.130	.718
rs35615695	2.916	.088

The results of chi‐square test indicated that the allelic and genotypic distributions of case and control groups had no obvious difference (for rs5005770, genotype: *χ* ^2^ = 4.782, *p *= .092, allele: *χ* ^2^ = .250, *p *= .617, OR = 0.929, 95% CI = 0.696–1.240; for rs12734374, genotype: *χ* ^2^ = 2.118, *p *= .146, allele: *χ* ^2^ = 2.086, *p *= .149, OR = 2.112, 95%CI = 0.748−5.962; for rs35615695, genotype: *χ* ^2^ = 4.144, *p *= .126, allele: *χ* ^2^ = .092, *p *= .762, OR = 0.958, 95% CI = 0.725−1.266). The results are shown in Table 3.

**TABLE 3:** The genotypic and allelic frequencies of three genetic loci in two groups

		rs5005770					rs12734374					rs35615695				
Group	N	GG	GA	AA	G	A	AA	AT	TT	A	T	TT	TC	CC	T	C
Patients	271	187	68	16	442	100	266	5	0	537	5	183	62	26	428	114
Control	337	217	108	12	542	132	324	13	0	661	13	219	99	19	537	137
*χ* ^2^		4.782			0.250		2.118			2.086		4.144			0.092	
*p*		.092			.617		.146			.149		.126			.762	
OR					0.929					2.112					0.958	
95%CI					0.696–1.240					0.748–5.962					0.725–1.266	

### 3.2 The family‐based study

The family‐based association test can further reveal the underlying genetic association after case–control test. The TDT test results showed a significant over‐transmission of alleles for rs35615695 (*χ* ^2^ = 57.375, *p *= .000, OR = 6.014, 95% CI = 3.657–9.978), while rs5005770 and rs12734374 indicated no significant over‐transmission (rs5005770, *χ* ^2^ = 1.065, *p* = .302, OR = 1.418, 95%CI = 0.729–2.759; rs12734374, *χ* ^2^ = .047, *p* = .828, OR = 0.991, 95%CI = 0.983−0.999). According to the results of HRR, rs5005770 tended towards a positive association with TS (*χ* ^2^ = 4.116, *p* = .042, OR = 1.483, 95%CI = 1.012–2.172) but failed to maintain significance after Bonferroni's correction (*p* = .017). In terms of HRR results, the rs12734374 and rs35615695 indicated no statistically significant evidence for an association with 271 TS trios (rs12734374, *χ* ^2^ = .000, *p* = 1.000, OR = 1.000, 95%CI = 0.286–3.494; rs35615695, *χ* ^2^ = 1.259, *p* = .262, OR = 1.234, 95%CI = 0.855−1.781). The results are shown in Tables 4 and 5.

**TABLE 4:** TDT results of three genetic loci in 271 trios

				TDT results			
			
SNP	Transmitted allele	Non‐transmitted allele		*χ* ^2^	*p*	OR	95%CI
rs5005770		G	A	1.065	.302	1.418	0.729−2.795
	G	389	41				
	A	87	13				
rs12734374		A	T	0.047	.828	0.991	0.983−0999
	A	532	5				
	T	532	0				
rs35615695		T	C	57.375	.000	6.041	3.657−9.978
	T	389	39				
	C	71	43				

**TABLE 5:** HRR results of three genetic loci in 271 trios

				HRR results			
			
SNP	Group	Transmitted allele	Non‐transmitted allele	*χ* ^2^	*p*	OR	95%CI
rs5005770	A(+)	84	63	4.116	.042	1.483	1.012−2.172
	A(–)	187	208				
rs12734374	T(+)	5	5	0.000	1.000	1.000	0.286−3.494
	T(–)	266	266				
rs35615695	C(+)	88	76	1.259	.262	1.234	0.855−1.781
	C(–)	183	195				

The HHRR analysis that enlarges the number of cases effectively is performed to improve the efficiency of the test. The positive results were found for rs5005770 and rs35615695 for HHRR analysis (rs5005770, *χ* ^2^ = 8.223, *p* = .004, OR = 0.613, 95%CI = 0.438–0.858; rs35615695, *χ* ^2^ = 4.807, *p* = .028, OR = 0.708, 95%CI = 0.520−0.965). Moreover, rs5005770 polymorphism maintained significance after Bonferroni correction (*p*  < .017), while the rs35615695 was negative. The rs12734374 also showed negative results for HHRR analysis (*χ* ^2^ = .000, *p* = 1.000, OR = 1.000, 95%CI = 0.288−3.474). All HHRR results are shown in Table 6.

**TABLE 6:** HHRR results of three genetic loci in 271 trios

				HHRR results			
			
SNP	Group	Transmitted allele	Non‐transmitted allele	*χ* ^2^	*p*	OR	95%CI
rs5005770	G	442	476	8.223	.004	0.613	0.438−0.858
	A	100	66				
rs12734374	A	537	537	0.000	1.000	1.000	0.288−3.474
	T	5	5				
rs35615695	T	428	456	4.807	.028	0.708	0.520−0.965
	C	114	86				

## 4 DISCUSSION

Over the past decade, studies that focused on the assessment of the impact of TS on health‐related quality of life have increased gradually. Almost all studies suggested a consistent decreased quality of life in TS patients (A. Cavanna et al., 2013; A. E. Cavanna et al., 2008; Eddy et al., 2011). We also investigated the quality of life of 107 TS patients and 107 healthy controls via the Inventory of Subjective Life quality and showed that TS had a severe influence on the patient's family life, school life, peer relationships, cognition, environmental awareness, self‐awareness, and depression (S. Liu et al., 2017). Under the circumstances, the effective therapeutic strategy of TS should be taken action to relieve the burden of TS patients and their families. However, there is no ideal anti‐tic treatment currently. When tics are at their worst, no drug or technique can be used effectively. Long‐term treatment was required when patients have taken most existed pharmacological drugs, and many of them may have clinically significant side effects. Consequently, it is highly beneficial to improve the understanding of TS pathogenesis for the development of new treatments.

Previous studies have presented that the ASH1L protein encoded by *ASH1L*, as a transcriptional activator, would result in epigenetic alternations in the humans. *ASH1L* is considered a highly credible autism susceptibility gene included in the Simons Foundation Autism Research Initiative gene database (https://gene.sfari.org/). Wang et al. (Wang et al., 2016) performed the gene mutation screening in 1543 cases of Chinese autistic population and revealed the variation (p.V2080I) in *ASH1L*. Shen et al. (Shen et al., 2019) detected a new frameshift mutation (p.Lys808TyrfsTer40) in *ASH1L* in a patient with multiple congenital anomalies (MCA), who presented with ADHD, learning disabilities, motor, and developmental delay. The follow‐up functional experiment confirmed the loss‐of‐function of ASH1L caused by the variants. Stessman et al. (2017) conducted targeted sequencing on 11,730 patients with neurodevelopmental disorders and found that *ASH1L* mutation was in all the patients with intellectual disabilities, two‐thirds of them with autism and two‐thirds with epilepsy, indicating that *ASHIL* may be involved in a variety of neurodevelopmental disorders.

We identified four transmitted variants (p.S74L, p.Y2077F, p.R1516C, and p.R2630T) and a de novo variant (p.K1547E) of *ASH1L* in TS patients among five families via conducting WES in 100 TS‐affected trios (S. Liu et al., 2020). Three variants (p.S74L, p.R1516C, and p.R2630T) have been reported in ASD cases (Stessman et al., 2017). Subsequently, targeted sequencing of *ASH1L* was performed in additional 524 unrelated TS samples and replicated the association. The enzymatic activity was affected by two‐point mutations (p.Y2077F and p.S2200G) (S. Liu et al., 2020). These rare variants played an important role in the pathogenesis of TS. We also constructed *ASH1L* ^+/–^ mouse model for further researches. Through observing the behavioral manifestations of *ASH1L* ^+/‐^ mice, we found that *ASH1L* ^+/−^ mice exhibited tic‐like behaviors, repetitive stereotype phenotypes, and abnormal social behaviors. In addition, compared with WT mice, the attack frequency in *ASH1L* ^+/−^ mice was significantly increased (S. Liu et al., 2020).

To further identify the common variants of *ASH1L* (rs5005770, rs12734374, and rs35615695) in the population, the combination of case–control and family‐based association studies was conducted in a large sample originating from Chinese Han. The TDT is a family‐based association study, which is not affected by population stratification, and provides a purer result or a lower false‐positive rate. The two methods have different strengths and limitations. When combined, the benefits of both led to avoid the interference of genetic factors and enhance the credibility of current research (Yang et al., 2018). In our study, TDT showed an obvious over‐transmission of the ASH1L rs35615695 polymorphism in TS. HHRR results also revealed a significant difference of rs5005770 and rs35615695 in family‐based study, especially rs5005770 maintaining significant after Bonferroni correction (*p *< .017). This evidence suggested that *ASH1L* may be a new risk gene for the development of TS.

However, the given experiments have some limitations. The current study was limited by the small sample size and race differences. Moreover, we only examined the associations of three SNPs with the risk of *ASH1L*, and the results may not be indicative of the entire association between *ASH1L* and TS. As a result, the association between ASH1L SNPs and TS should be investigated in a larger sample size deriving from different ethnicities. Due to the limitations of sample size, ethnic differences, and test standards, traditional case–control association analysis often yielded inconsistent experimental results, which were not sufficient to fully reveal the susceptibility genes of TS. TDT that based on the patient's biological parents as the research object is a kind of association studies based on family and to investigate whether there is linkage disequilibrium between alleles that are transmitted to affected offspring and those that are not. This method is not affected by population stratification and provides more “pure” results than case–control association analysis. Furthermore, the false‐positive rate is low. As the two research methods have their own advantages and limitations, the best method is to combine them, which can not only avoid the interference of genetic factors but also improve the statistical strength of the research.

Previously, the association analysis study of the candidate genes of CHAT (rs100824791 rs12264845, rs1880676, rs3793790, and rs3793798) was innovatively performed via a combination of traditional case–control analysis and family‐based association analysis in 400 cases of TS core family and 401 cases of healthy controls. Family‐based analysis of TDT, HRR, and HHRR showed that CHATrs3793790 was associated with TS, whereas case–control analysis showed the opposite (Yang et al., 2018). The candidate genes of SLC5A7 (rs1013940, rs2433718, and rs4676169) were analyzed with the same methods, and the results of the two methods showed that SLC5A7 had no significant association with TS (W. Liu et al., 2017).

In addition, as TS patients may be associated with neuronal development and dysfunction, we carried out a combination of traditional case–control analysis and family‐based association analysis for candidate genes related to neuronal development. The results showed that PCNTrs2839227 showed significant differences in case–control association analysis and family‐based association analysis (W. Liu et al., 2020). It is suggested that this gene may be associated with the disease of TS. Therefore, the combination of the two methods can significantly improve the testing strength and reliability of association analysis research methods.

To our knowledge, we first reported the association between *ASH1L* and TS in a Han Chinese population. The main aim of our research is to screen and identify the candidate susceptibility gene for TS. The identification of susceptibility genes relevant to the disease is an important step toward broadening our knowledge of TS pathogenesis and seeking for new therapeutic interventions or biomarker targets.

## AUTHOR CONTRIBUTIONS

Xueping Zheng and Fengyuan Che designed the project conception. Wenmiao Liu, Lulu Xu, Cheng Zhang, Lu Shen, Jicheng Dong, Han Zhang, and Shiguo Liu performed the literature search and conducted the experiment. Wenmiao Liu and Lulu Xu wrote the manuscript with contribution from Xueping Zheng and Fengyuan Che. All authors read and approved the final version of the manuscript.

## Data Availability

The data that support the findings of this study are available on request from the corresponding author.
